# Lower limb neuromotor control during perturbed and unperturbed gait conditions in male runners with Achilles tendinopathy: an exploratory analysis

**DOI:** 10.1080/23335432.2025.2611710

**Published:** 2026-01-05

**Authors:** Andrew Quarmby, Philip Kurtz, Mina Khajooei, Myoung-Hwee Kim, Michael Cassel, Frank Mayer, Tilman Engel

**Affiliations:** a Sport and Health Sciences, University of Potsdam, Potsdam, Germany; b University Outpatient Clinic, Sports Medicine & Sports Orthopaedics, Potsdam, Eugene, Oregon, Germany; c Department of Human Physiology, University of Oregon, USA

**Keywords:** EMG, Achilles tendinopathy, gait, runners, motor control

## Abstract

Achilles tendinopathy (AT) is common among runners and typically presents with Achilles tendon pain. Neuromotor alterations have been reported in AT during demanding tasks such as running; however, alterations during functional tasks such as walking remain poorly understood. Such information may be relevant and inform rehabilitation targets. Therefore, this exploratory study investigated lower limb neuromotor control during walking and running in people with AT compared with healthy controls (CO). Twenty-four male runners participated (AT = 12, CO = 12) and completed walking (WALK), perturbed walking (PERTWALK), running (RUN), and perturbed running (PERTRUN) on a custom split-belt treadmill. Lower limb muscle activity was recorded using electromyography to assess activity onset, offset, and duration during unperturbed tasks, and reflex latency and amplitude during perturbed tasks. A MANOVA (α = 0.05) examined group effects (AT/CO). During WALK, AT showed delayed onset of tibialis anterior, peroneus longus, and vastus medialis (p < 0.05). During RUN, gastrocnemius medialis duration was longer, while gluteus maximus onset was delayed and shorter in AT (p < 0.05). During PERTWALK, tibialis anterior reflex latency was delayed in AT (p < 0.05), whereas reflex amplitudes and all PERTRUN outcomes were not significant. These findings indicate potential neuromotor differences in AT.

## 1. Introduction

Achilles tendinopathy (AT) is a musculoskeletal condition, presenting clinically with Achilles tendon pain, morning stiffness, increased tendon thickness, and associated tissue degeneration (Cook et al. 2016; de Vos et al. 2021; Scott et al. 2020). AT is known to occur in both athletes and the general population (de Jonge et al. 2011). The lifetime incidence in running athletes is reported between 24% and 52% (Kujala et al. 2005; Munteanu and Barton 2011), and the condition is usually longstanding, typically leading to reduced participation in sport, and disability (de Jonge et al. 2011; de Vos et al. 2021).

There are a number of reported risk factors associated with AT (van der Vlist et al. 2019), amongst them altered lower limb biomechanics during dynamic motor tasks (Ogbonmwan et al. 2018; Sancho I, Malliaras P, et al. 2019). Specifically, neuromotor control has been investigated in several studies, with results indicating that persons with AT exhibit distinct movement characteristics when compared with healthy controls (Baur et al. 2011; Franettovich et al. 2014; Hein et al. 2014; Chang and Kulig 2015; Creaby et al. 2017; O’Neill et al. 2019; Sancho et al. 2022). For example, persons with AT display altered electromyographic (EMG) profiles during tasks such as running, including reduced amplitudes of the gastrocnemius musculature during weight acceptance and push-off (Baur et al. 2004, 2011), an earlier offset of the soleus muscle (Wyndow et al. 2013), and delayed onset of the gluteal muscles (Franettovich et al. 2014). Additionally, during sub-maximal hopping, the contribution of the primary plantarflexors is minimised in people with AT, and accordingly compensated for by elevated peroneus longus activity (Chang and Kulig 2015). Furthermore, an investigation of such alterations during walking tasks appears to be mostly unexplored (Quarmby et al., 2023).

Overall, it seems appropriate to assume that neuromotor behaviour is altered in persons with AT; however, the reasons and mechanisms for this phenomenon are not well understood. It is commonly proposed that people with persistent pain disorders move differently (Hodges and Tucker 2011), probably as an adaptive response to avoid further perceived ‘damage’ to the painful tissue. In addition, research has shown that the mechanical properties of a tendinopathic Achilles tendon are altered (Arya and Kulig 2010), whereby the tendon displays reduced mechanical stiffness, thus negatively impacting the viscoelastic performance properties of the tendon (Child et al. 2010; Intziegianni et al. 2016). It has been speculated that these local mechanical alterations in AT patients result in the persistent adaptations in lower limb neuromotor control that seem evident in the literature (Kulig et al. 2020), perhaps due to self-regulative adjustments throughout the kinetic chain (Karandikar and Vargas 2011). However, it should be noted, that not all AT patients reach such a degenerative stage of tendinopathy (Cook et al. 2016), and central pain processing also appears changed in people with AT (Tompra et al. 2016) which will likely contribute to the persistence of symptoms and the employed motor strategies of individuals with the condition (Hodges and Tucker 2011).

Neuromuscular reflex responses have received research attention in other areas of musculoskeletal medicine, most notably in chronic low back pain (Liebetrau et al. 2013). Reflex responses seem to be delayed in back pain patients (Mueller et al. 2017), and alterations are also seen in patients with chronic ankle instability (Lin et al. 2021). The study of neuromotor responses to sudden and unexpected perturbations, provides a window to investigate the integrity of the sensory motor system in the presence of pathology. Experimental data from two studies in AT patients indicate that reflexes are upregulated in the triceps surae musculature (Wang et al. 2011; Chang and Kulig 2015). However, this research utilized artificial electrical stimulation to elicit reflex responses, rather than examining neuromuscular activity naturally evoked during functional tasks such as walking or running. More ecologically valid methods are likely to provide more practical insights, particularly given that Achilles tendinopathy is highly prevalent among running athletes. It is therefore logical to study neuromotor behaviour within this specific context. Additionally, previous research has typically studied the motor control of independent joints such as the ankle and hip during gait tasks, whereas a comprehensive approach measuring the whole lower limb might allow for simultaneous analysis and comprehension. Insights from such research could then be directly applied in rehabilitative settings with the AT population, for example in the development of specific training programs.

Despite growing evidence that Achilles tendinopathy may involve neuromotor adaptations, it remains unclear whether these deficits extend to walking and running, particularly under conditions that challenge motor control. Prior studies have not comprehensively examined EMG-derived motor control strategies across both steady-state and perturbed gait. By filling this gap, the present study provides novel mechanistic insights that can refine theoretical models of tendon-related neuromotor adaptation and generate hypotheses for future investigations into targeted rehabilitation and injury-prevention strategies.

Therefore, the aims of this exploratory study were twofold: 1) To investigate lower limb neuromotor control during unperturbed walking and running in AT patients compared with healthy controls, 2) To investigate lower limb neuromotor reflex responses during perturbed walking and running in people with AT compared with asymptomatic controls. Based upon current literature, it was hypothesised that: 1) Timing of the neuromotor signal would be early in the triceps surae musculature and delayed in the gluteal muscles in people with AT, 2) Reflex latencies would be delayed in response to the perturbations during gait in persons with AT, 3) Reflex amplitudes would be reduced in the triceps surae musculature in the AT group.

## 2. Materials and methods

### 2.1. Participants

A convenience sample of 24 individuals participated in the current study, including 12 participants with mid-portion Achilles tendinopathy (AT) and 12 asymptomatic controls. Participants were recruited from local sport clubs, via social media advertising, and word of mouth. Ethical approval was granted by the local University of Potsdam ethics commission (application number 74/2021), and all participants provided informed consent. Inclusion criteria for all participants were to be aged between 18 and 65 years old, male, and to be involved in a running sport for ≥20 km per week. Participants in both groups were excluded if they had any cardiovascular, neurological, or musculoskeletal injury, surgery or condition (apart from AT) within the last 6 months. Further exclusion criteria were acute infection, previous Achilles tendon rupture, diagnosis of insertional tendinopathy, and pain in the Achilles tendon during loading tasks ( > 4/10, NRS). To be included in the AT group, individuals were diagnosed by a medical doctor based upon criteria published from Hutchison et al. (2013). Criteria were defined as: 1) History of pain for at least three months local to the mid-portion of the Achilles tendon (2–7 cm proximal to the insertion), 2) Pain on palpation of the Achilles tendon mid-portion. Ultrasonographic imaging of the tendons was additionally performed in all participants [Canon Xario 200 G (CUS-X200G), 50-60 Hz], with measurements taken at the thickest part of the Achilles tendon. Ultrasound data was used to quantify tendon thickness diameter and observable tissue alterations indicative of tendinosis (hypo- and hyperechogenicities as well as neovascularization) but as per recent guidelines, was not explicitly utilized to inform a diagnosis of AT (de Vos et al. 2021).

### 2.2. Questionnaires

Data was collected on participant’s age (years), weight (kg), and height (cm). Additionally, participants were requested to fill out the International Physical Activity Questionnaire – Short Form (IPAQ) (Lee et al. 2011), and distance ran per week (km) was subjectively recalled. Participants also completed the Victorian Institute of Sport Assessment Achilles questionnaire (VISA-A) (Robinson et al. 2001) to measure clinical severity of AT, and were asked to recall symptoms duration (months). Finally, participants were asked to rate the level of pain in their Achilles tendon on a Numerical Rating Scale (0–10, NRS) (Williamson and Hoggart 2005) both at rest and during lower limb physical activity, e.g. hopping.

### 2.3. Measurements

After the clinical examination and questionnaires, participants were prepared for the subsequent trials. Data was collected in the dominant leg of the control group, and in the symptomatic or most symptomatic leg (in bilateral cases) of the AT group. To measure muscle activity, electromyography (EMG) was applied in seven muscles of the lower limb. Bipolar EMG electrodes [2 cm inter-electrode distance, pre-gelled (Ag/AgCl), type *p*-00-S, Ambu, Mediocotest, Denmark] were placed at the M. tibialis anterior (TA), M. peroneus longus (PL), M. soleus (Sol), M. gastrocnemius medialis (GM), M. vastus medialis (VM), M. biceps femoris (BF), and M. gluteus maximus (Gmax). Electrodes were positioned with reference to the SENIAM guidelines (Hermens et al. 2000). A wireless EMG recording system (band-pass filter: 5–500 Hz, gain: 5.0, overall gain: 2500, sampling frequency: 4000 Hz; Myon320, RFTD-32, myon AG, Switzerland) was utilized for data capture. Furthermore, an accelerometer (ACC) (Myon320s, myon AG, Switzerland) was attached to the heel of each shoe, to measure foot position and velocity, enabling identification of gait phases.

### 2.4. Protocol

Participants wore standardised shoes (Nike, Pegasus), for the duration of the trial. Initially, a warm-up was performed on a step for 60 s, in which time the signal quality of the EMG and ACCs was checked. Participants then walked (WALK) for 3 min at 3.6 km/h on a flat-grade treadmill [Woodway, Germany; for technical details see: (Quarmby et al. 2020)]. After a short break, the perturbed walking trial (PERTWALK) began, whereby participants walked at the same baseline velocity (3.6 km/h) and 10 superimposed perturbations were executed via the treadmill belts on both the left and right side. This protocol and its reliability has been described thoroughly in previous work (Engel et al. 2017), but essentially the decelerative treadmill belt impulses were delivered rapidly and powerfully in the mid-stance of gait, resulting in a stumbling event and provoking neuromuscular reflex responses during locomotion. Participants were instructed to recover their gait and continue walking as normal. Next, the running trials began, commencing with 3 min of unperturbed running (RUN) at 9 km/h. Following this, participants were asked to complete the perturbed running trial (PERTRUN), in which 15 superimposed perturbations occurred on the right and left side, at a baseline velocity of 9 km/h. This protocol has been described in detail beforehand (Quarmby et al. 2020), and similar to the perturbed walking protocol aims to disturb participants in the mid-stance of running gait with powerful decelerative belt impulses which result in a neuromuscular reflex response. The perturbations were randomized by time and side, ensuring that the delivered belt impulses were unexpected. Participants were asked to rate their levels of pain (NRS, 0–10) in the Achilles tendon during each of the four independent trials. Moreover, the running style of each person was subjectively assessed and categorized as either ‘rearfoot’ or ‘forefoot’.

### 2.5. Data analysis

Anthropometrics, questionnaire, and pain data were analysed descriptively (mean±SD). Ultrasound data of the Achilles tendon were analysed by a medical examiner at time of assessment. An Achilles tendon diameter of ≥7 mm was classified as ‘thickened’ and tendons displaying signs of tendinosis with visible hypoechogenicity of the signal were classified as ‘degenerative’ (Docking et al. 2015; Cassel et al. 2017). The data from the IPAQ was analysed based upon established guidelines (Lee et al. 2011), resulting in an output of physical activity measured in metabolic equivalent minutes per week (MET min/Week).

**Figure 1.**
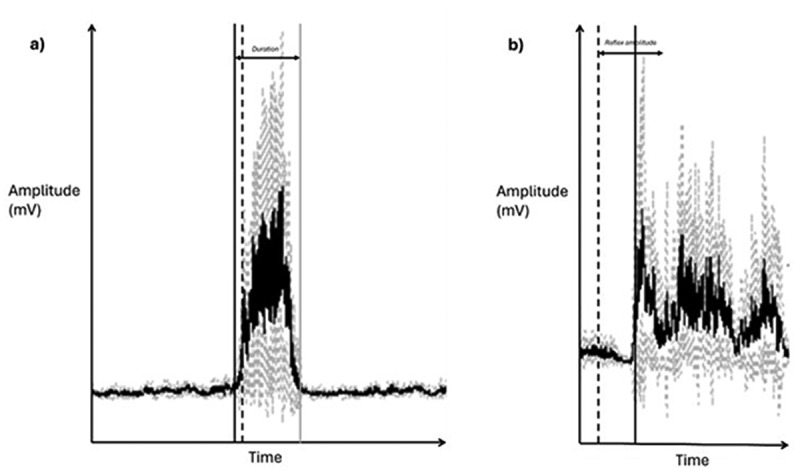
Example of rectified electromyographic signal in two different tasks, the mean of the signal is shaded dark, and the lighter shaded signal represents standard deviation. Left figure: a) the gastrocnemius medialis muscle during unperturbed walking. The solid black line represents time of muscle onset, the dashed black line is initial ground contact, and the solid grey line represents time of muscle offset. Duration of the muscle activation is time between onset and offset. Right figure: b) the tibialis anterior muscle during perturbed walking. The dashed black line represents time of perturbation onset, the solid black line is time of muscle onset (reflex latency). Reflex amplitude is indicated as the solid black line with two arrows at either end, representing an approximate window of 200 ms along the x-axis of time.

EMG and ACC data were synchronised and analysed in the same software (4th order moving average filter, IMAGO process master, pfitec, biomedical systems, Germany). EMG data were full wave rectified and checked manually for artefacts. For the unperturbed WALK and RUN trials, initial ground contact was identified using ACC data (vertical Z axis), based on visually identifiable signal spikes corresponding to the first detectable deceleration after foot swing. This process was repeated for 10 consecutive strides. An ensemble average of the unperturbed gait data was then produced to identify three temporal EMG variables (see Figure 1a): 1) *Onset* – timepoint at which the muscle is active (ms), 2) *Offset* – timepoint at which muscle stops being active (ms), 3) *Duration* – total duration of muscle activity (ms). A muscle was considered to be ‘on’ when the EMG amplitude was two standard deviations (SD) above the resting signal for at least 100 ms (Baur et al. 2011; Wyndow et al. 2013), calculated via semi-automated detection and visually confirmed in the data. Onset and offset were then accordingly defined as the time (ms) at which the muscle was active in relation to initial contact (Franettovich et al. 2014). Duration was defined as the total length of time that the muscle was active for (the difference between the onset and offset times).

For the perturbed trials of PERTWALK and PERTRUN, perturbation initiation was identified in the ACC data in both trials. An ensemble average of all identifiable perturbations was then created for each trial, to enable assessment of two further variables (see Figure 1b): 1) the temporal variable *reflex latency –* time taken for the muscle to be active in response to the perturbation (ms), and 2) *reflex amplitude –* the magnitude of the EMG signal in response to the perturbation when normalised to the same phase of unperturbed gait (%). Reflex latency was calculated as the time between perturbation initiation and onset of the EMG signal at an amplitude of 2SD above the resting signal (ms). Reflex amplitude was assessed by calculating the root mean square and normalising the average activity of 200 ms time windows post-perturbation initiation, to 200 ms time windows commencing 200 ms (WALK) and 150 ms (RUN) after heel strike during unperturbed gait (Taube et al. 2007; Engel et al. 2017). For purposes of nomenclature: the variable ‘onset’ was considered as feedforward motor behaviour, whereas the variables ‘reflex latency’ and ‘reflex amplitude’ were defined as reactive motor behaviour. Stride time (sec) was determined via the ACC data in the unperturbed WALK and RUN trials, defined as the time taken from initial contact to subsequent initial contact in the same foot, calculated as an average of 10 consecutive strides.

### 2.6. Statistical analysis

Data were analysed in Excel (Microsoft Corporation 2021) and SPSS Statistics (22, IBM Corporation, Armonk, NY, USA). Data were normally distributed. To analyse for differences between-groups, separate multi-variate ANOVAs were performed for each muscle and task type (walking and running). Assumptions required for MANOVA were evaluated, including multivariate normality and homogeneity of covariance matrices. These assumptions were largely met; however, given the small sample size, tests of assumptions should be interpreted cautiously. Tests for the temporal variables of onset, offset, duration, and reflex latency were conducted independently from the tests for the magnitude variable of reflex amplitude. The MANOVAs were performed with the independent variable set as the between-subject factor of group (Achilles tendinopathy (AT), control (CO)), followed up with univariate testing for specific interpretation. An *α* level of 0.05 was set for statistical tests. No post hoc corrections for multiple comparisons were applied in order to retain statistical power and avoid Type II errors, given the exploratory nature of the study. Therefore, results should be interpreted with caution and viewed as preliminary (Bender and Lange 2001). Correspondingly, mean±SD, and lower and upper 95% confidence intervals (CI) were reported for each group, alongside p-values for tests between groups. Additionally, effect sizes were calculated for all variables and muscles between groups (Cohen’s *d* = (*M*_2_ - *M*_1_) ⁄ *SD*_pooled_). Cohen’s d values were interpreted as 0.2 to 0.5 (small), 0.5 to 0.8 (medium) and ≥0.8 (large) (Hopkins 2000).

## 3. Results

### 3.1. Participant characteristics

Anthropometrics, questionnaire, and baseline pain data are displayed in Table 1. The AT group was on average 5 years older than the CO group. Most participants in both groups scored in the ‘high’ domain for physical activity according to the IPAQ, with the AT group being slightly more active on average. Ten out of 12 (83%) AT participants showed thickening and degenerative changes in the Achilles tendon, whereby the two participants not displaying identifiable structural pathology had only had symptoms for around 12 months. The AT group scored an average of 73 ± 11 on the VISA-A.

**Table 1.** Participant characteristics.

	AT (*n* = 12)	CO (*n* = 12)
Age (years)	42 ± 9	37 ± 7
Height (cm)	182 ± 6	181 ± 6.5
Weight (kg)	79 ± 10	77 ± 5
IPAQ (MET mins per week)	4481 ± 3014	3977 ± 2215
Distance ran per week (km)	31 ± 13	28 ± 15
VISA-A	73 ± 11	98 ± 2
Duration of symptoms (months)	52 ± 52	–
Pain in tendon at rest (NRS, 0–10)	0.6 ± 0.8	0.0 ± 0.0
Pain in tendon during exercise (NRS, 0–10)	2.9 ± 1.1	0.0 ± 0.0
Tendon thickening (%)	83%	0%
Degenerative changes in tendon (%)	83%	8%
Forefoot runners (%)	25%	17%

### 3.2. Stride time

Stride time was similar between both groups in both tasks (WALK: AT = 1.18 ± 0.08 sec, CO = 1.22 ± 0.07 sec; RUN: AT = 0.77 ± 0.03 sec, CO = 0.77 ± 0.05 sec).

### 3.3. Temporal variables

The multi-variate ANOVAs indicated statistically significant main effects in temporal variables for the muscles TA during walking (*p* = 0.02), and GM during running (*p* = 0.05). The remaining tests of main effects were statistically insignificantfor example, PL in walking (*p* = 0.08) and Gmax in running (*p* = 0.09).

### 3.4. EMG: unperturbed gait

**Figure 2.**
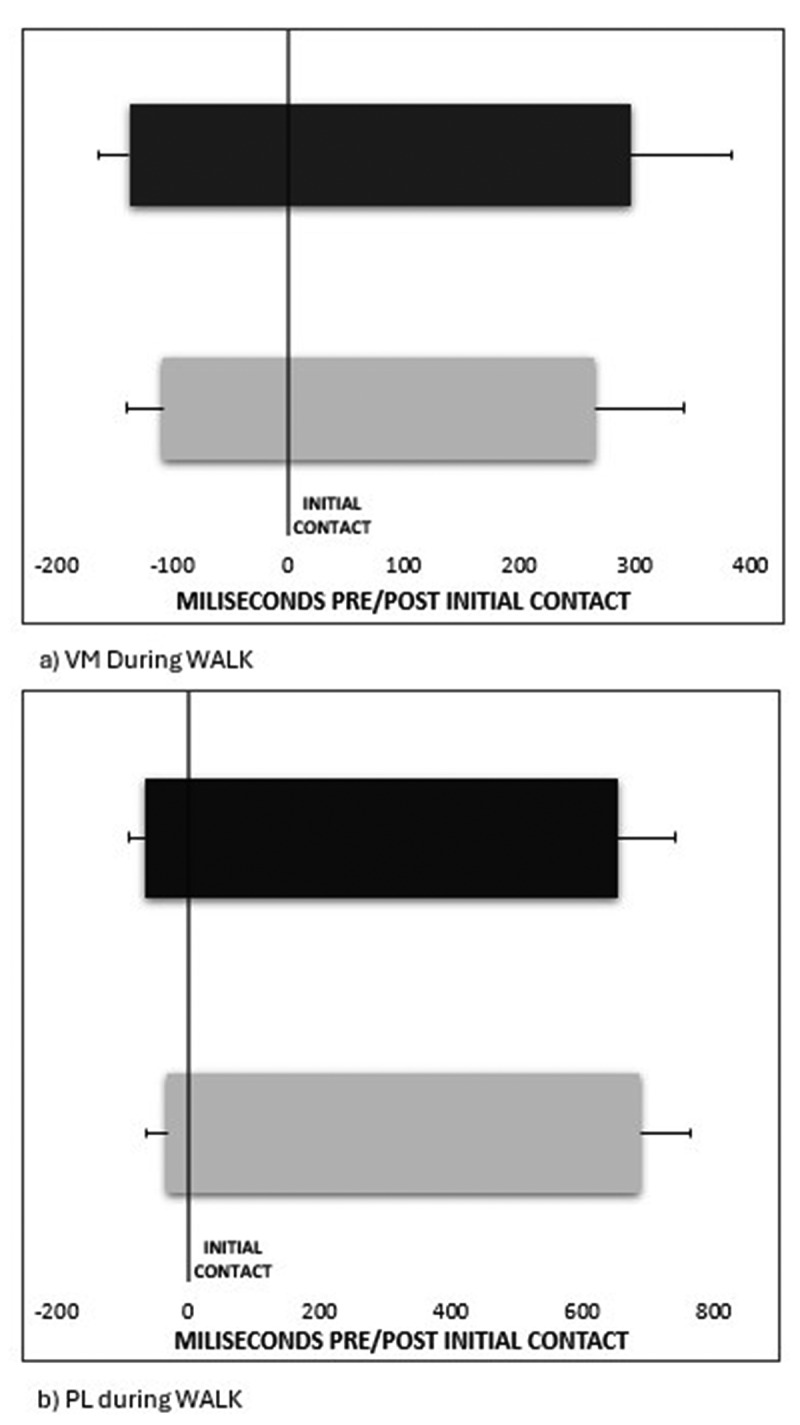
EMG muscle activity (mean±SD) during unperturbed walking: a) vastus medialis, b) peroneus longus. Data for AT are coloured grey, and control are black. The vertical black line indicates initial contact during gait. Negative values indicate EMG onset (prior to initial contact), and positive values are EMG offset (after initial contact).

**Figure 3.**
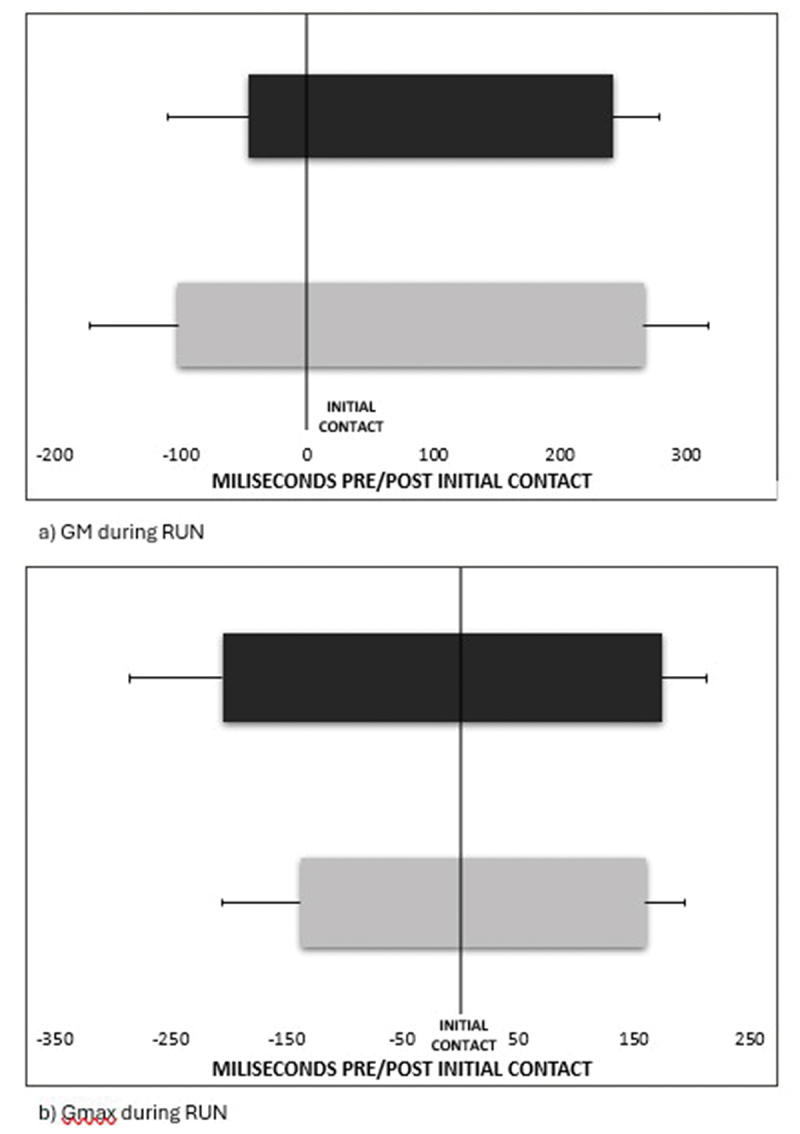
EMG muscle activity (mean±SD) during unperturbed running: a) gastrocnemius medialis muscle, b) gluteus maximus muscle. Data for AT are coloured grey, and control are black. The vertical black line indicates initial contact during gait. Negative values indicate EMG onset (prior to initial contact), and positive values are EMG offset (after initial contact).

The three temporal variables analysed during unperturbed gait of onset, offset, and duration are reported in Table 2 for both walking and running tasks. Between-subject differences were found for TA onset during WALK, which was delayed in the AT group (*p* = 0.02, Cohen’s d = 1.03). Additionally, during WALK, PL onset (*p* = 0.01, Cohen’s d = 1.18) and VM onset (*p* = 0.03, Cohen’s d = 0.98) were significantly delayed in the AT group (see Figure 2). The magnitude of difference during WALK of PL and VM onset represents approximately 3% of the gait cycle. In RUN, GM duration was significantly longer in the AT group (*p* = 0.01, Cohen’s d = 1.32), and GM onset appeared early in the AT group, though only approaching statistical significance (*p* = 0.06, Cohen’s d = 0.83) (see Figure 3). The between-group differences in GM onset and duration, represent 7% and 9% of the gait cycle respectively. Moreover, during RUN, Gmax onset was delayed (*p* = 0.04, Cohen’s d = 0.9) and Gmax duration was shorter (*p* = 0.03, Cohen’s d = 0.98) in the AT group (see Figure 3). As a proportion, these between group differences represent 9% and 11% of the gait cycle.

**Table 2.** Data for the temporal EMG variables during unperturbed and perturbed walking and running.

WALK	Mean ± standard deviation	Lower and upper 95% CI	*P*-Value	Effect size (Cohen’s d)	RUN	Mean ± standard deviation	Lower and upper 95% CI	*P*-Value	Effect size (Cohen’s d)	
***M.*** ***Tibialis anterior***					***M. Tibialis anterior***					
Onset (ms)					Onset (ms)					
AT	−436 ± 67	[−471, −400]	**0.021**	1.03	AT	−432 ± 67	[−465, −399]	0.188		0.57
CO	−496 ± 50	[−531, −461]			CO	−463 ± 40	[−496, −430]			
Offset (ms)					Offset (ms)					
AT	339 ± 192	[216, 463]	0.77	0.12	AT	185 ± 94	[138, 232]	0.833		0.09
CO	315 ± 219	[191, 439]			CO	178 ± 59	[131, 225]			
Duration (ms)					Duration (ms)					
AT	776 ± 181	[651, 901]	0.682	0.17	AT	616 ± 96	[571, 662]	0.436		0.34
CO	811 ± 233	[686, 936]			CO	641 ± 48	[595, 686]			
Reflex latency (ms)					Reflex latency (ms)					
AT	96 ± 6	[93, 100]	**0.017**	1.06	AT	115 ± 7	[109, 121]	0.797		0.1
CO	90 ± 6	[87, 94]			CO	114 ± 12	[108, 120]			
***M.*** ***Peroneus longus***					***M. Peroneus longus***					
Onset (ms)					Onset (ms)					
AT	−31 ± 32	[−48, −14]	**0.009**	1.18	AT	−71 ± 34	[−91, −52]	0.550		0.25
CO	−64 ± 25	[−82, −48]			CO	−63 ± 32	[−83, −44]			
Offset (ms)					Offset (ms)					
AT	688 ± 133	[604, 774]	0.558	0.24	AT	225 ± 44	[161, 289]	0.469		0.34
CO	654 ± 150	[569, 739]			CO	257 ± 144	[193, 321]			
Duration (ms)					Duration (ms)					
AT	720 ± 136	[635, 804]	0.991	0.01	AT	297 ± 47	[234, 359]	0.579		0.26
CO	719 ± 146	[635, 803]			CO	320 ± 139	[258, 383]			
Reflex latency (ms)					Reflex latency (ms)					
AT	97 ± 8	[87, 109]	0.743	0.15	AT	–	–	–		–
CO	100 ± 25	[89, 112]			CO	–	–	–		–
***M.*** ***Soleus***					***M. Soleus***					
Onset (ms)					Onset (ms)					
AT	−7 ± 53	[−52, 38]	0.179	0.59	AT	−77 ± 48	[−106, −47]	0.155		0.6
CO	35 ± 92	[−10, 80]			CO	−47 ± 50	[−77, −17]			
Offset (ms)					Offset (ms)					
AT	700 ± 133	[618, 783]	0.581	0.23	AT	252 ± 44	[224, 280]	0.758		0.15
CO	669 ± 142	[586, 751]			CO	247 ± 44	[219, 274]			
Duration (ms)					Duration (ms)					
AT	708 ± 124	[625, 790]	0.201	0.54	AT	329 ± 51	[296, 361]	0.080		0.75
CO	633 ± 150	[551, 716]			CO	294 ± 43	[266, 320]			
Reflex latency (ms)					Reflex latency (ms)					
AT	–	–	–	–	AT	–	–	–		–
CO	–	–	–	–	CO	–	–	–		–
***M.*** ***Gastrocnemius medialis***					***M. Gastrocnemius medialis***					
Onset (ms)					Onset (ms)					
AT	64 ± 63	[20, 110]	0.368	0.38	AT	−102 ± 70	[−146, −57]	0.055		0.83
CO	93 ± 84	[48, 138]			CO	−46 ± 65	[−87, 5]			
Offset (ms)					Offset (ms)					
AT	619 ± 102	[548, 691]	0.742	0.14	AT	266 ± 51	[233, 298]	0.208		0.42
CO	635 ± 134	[564, 707]			CO	242 ± 37	[219, 265]			
Duration (ms)					Duration (ms)					
AT	554 ± 89	[486, 623]	0.804	0.1	AT	368 ± 67	[325, 410]	**0.006**		1.32
CO	543 ± 135	[474, 611]			CO	298 ± 42	[272, 326]			
Reflex latency (ms)					Reflex latency (ms)					
AT	–	–	–	–	AT	–	–	–		–
CO	–	–	–	–	CO	–	–	–		–
***M.*** ***Vastus medialis***					***M. Vastus medialis***					
Onset (ms)					Onset (ms)					
AT	−108 ± 32	[−126, −91]	**0.026**	0.98	AT	−91 ± 26	[−111, −72]	0.141		0.63
CO	−137 ± 26	[−154, −119]			CO	−111 ± 37	[−131, −92]			
Offset (ms)					Offset (ms)					
AT	265 ± 77	[215, 315]	0.36	0.38	AT	173 ± 44	[148, 198]	0.526		0.26
CO	296 ± 87	[247, 345]			CO	184 ± 40	[159, 209]			
Duration (ms)					Duration (ms)					
AT	373 ± 95	[318, 428]	0.123	0.65	AT	264 ± 59	[224, 304]	0.267		0.47
CO	433 ± 87	[378, 487]			CO	295 ± 74	[255, 335]			
Reflex latency (ms)					Reflex latency (ms)					
AT	108 ± 12	[96, 120]	0.458	0.33	AT	–	–	–		–
CO	113 ± 26	[102, 126]			CO	–	–	–		–
***M.*** ***Biceps femoris***					***M. Biceps femoris***					
Onset (ms)					Onset (ms)					
AT	−204 ± 80	[−245, −164]	0.923	0.04	AT	−215 ± 55	[−245, −184]	0.936		0.03
CO	−202 ± 50	[−242, −161]			CO	− 216 ± 46	[−247, −186]			
Offset (ms)					Offset (ms)					
AT	260 ± 122	[166, 354]	0.867	0.07	AT	242 ± 49	[207, 277]	0.157		0.6
CO	249 ± 184	[156, 343]			CO	207 ± 67	[172, 242]			
Duration (ms)					Duration (ms)					
AT	464 ± 98	[401, 526]	0.803	0.09	AT	457 ± 62	[415, 494]	0.262		0.51
CO	450 ± 208	[318, 583]			CO	423 ± 70	[384, 463]			
Reflex latency (ms)					Reflex latency (ms)					
AT	81 ± 11	[72, 89]	0.294	0.44	AT	–	–	–		–
CO	74 ± 15	[66, 83]			CO	–	–	–		–
***M.*** ***Gluteus maximus***					***M. Gluteus maximus***					
Onset (ms)					Onset (ms)					
AT	−79 ± 37	[−98, −59]	0.242	0.5	AT	−138 ± 68	[−183, −94]	**0.039**		0.9
CO	−95 ± 27	[−114, −75]			CO	−205 ± 80	[−249, −160]			
Offset (ms)					Offset (ms)					
AT	299 ± 138	[228, 371]	0.224	0.52	AT	160 ± 34	[139, 182]	0.365		0.38
CO	238 ± 98	[167, 310]			CO	174 ± 39	[152, 196]			
Duration (ms)					Duration (ms)					
AT	378 ± 120	[308, 448]	0.358	0.38	AT	298 ± 85	[249, 348]	**0.026**		0.98
CO	333 ± 115	[263, 403]			CO	379 ± 80	[330, 428]			
Reflex latency (ms)					Reflex latency (ms)					
AT	146 ± 38	[125, 167]	0.323	0.41	AT	–	–	–		–
CO	160 ± 33	[139, 181]			CO	–	–	–		–

### 3.5. EMG: perturbed gait

**Figure 4.**
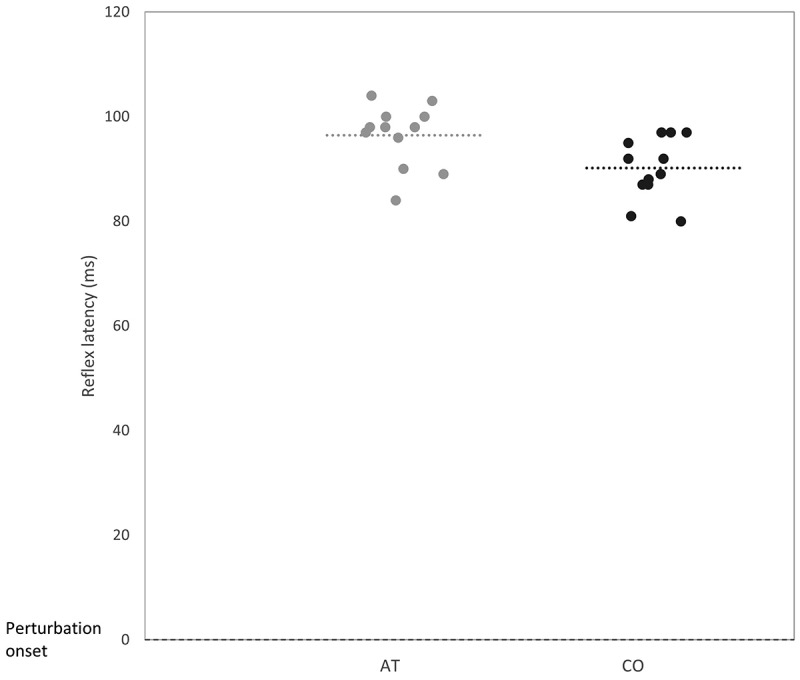
Reflex latencies of the tibialis anterior muscle during perturbed walking. The x-axis indicates time the perturbation hit (dashed horizontal line) and group membership. The y-axis indicates time taken for the EMG signal to be identifiable (reflex latency). Each dot in the scatterplot represents the mean of a single participant. The grey colour represents the AT group, and the black colour is CO. The dashed line signifies the mean of each group.

During PERTWALK, reflex latencies of the GM and Sol could not be assessed, due to artefacts in the EMG signal. In the PERTRUN task, only the TA muscle could be included for reflex latency analysis, due to difficulties in identification of a resting EMG signal. Significant between-group differences were found only in the reflex latency of the TA muscle during PERTWALK (*p* = 0.02, Cohen’s d = 1.06), displayed in Table 2 and Figure 4.

### 3.6. Reflex amplitudes

**Figure 5.**
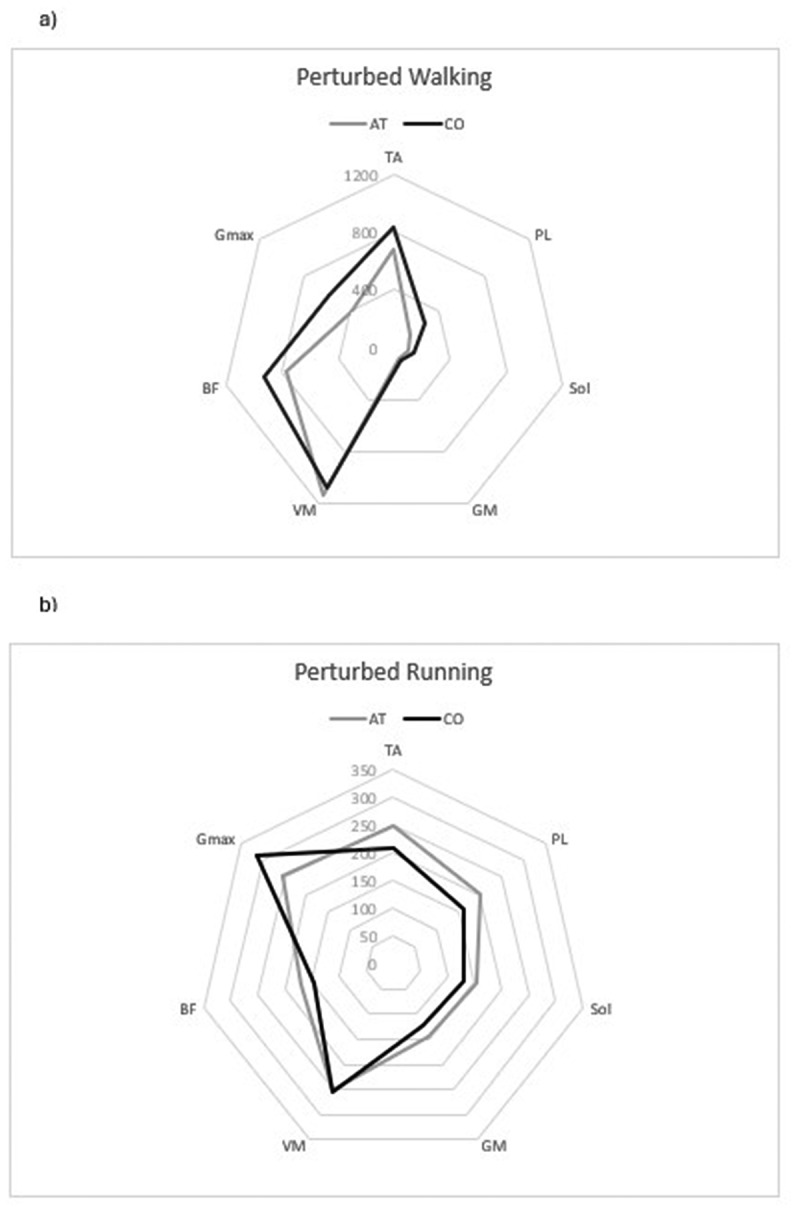
Reflex amplitudes (%) of the lower limb muscles in response to perturbations in: a) perturbed walking, b) perturbed running. Values further from the centre of the radar plot represent higher levels of EMG muscle activity.

Regarding reflex amplitudes in response to the perturbations, there were no statistically significant differences found between-groups, in both PERTWALK and PERTRUN (see Table 3 and Figure 5). This was true for the main effects and in specific between-subject effects of each dependent variable. However, there was a trend in between-subject effects, indicating reduced reflex amplitudes in PL (*p* = 0.06, Cohen’s d = 0.93), Sol (*p* = 0.07, Cohen’s d = 0.79) and GM (*p* = 0.07, Cohen’s d = 0.78) in the AT group during PERTWALK.

**Table 3.** Data for EMG reflex amplitudes during perturbed walking and running.

WALK	Mean ± standard deviation (%)	Lower and upper 95% CI (%)	*P*-value	Effect size (Cohen’s d)	RUN	Mean ± standard deviation (%)	Lower and upper 95% CI (%)	*P*-value	Effect size (Cohen’s d)
***M.*** ***Tibialis anterior***					***M. Tibialis anterior***				
AT	678 ± 359	[450, 907]	0.281	0.45	AT	249 ± 115	[176, 321]	0.273	0.49
CO	831 ± 317	[630, 1032]			CO	208 ± 51	[175, 240]		
***M.*** ***Peroneus longus***					***M. Peroneus longus***				
AT	147 ± 68	[104, 190]	0.055	0.93	AT	200 ± 89	[142, 256]	0.204	0.55
CO	277 ± 213	[143, 413]			CO	160 ± 57	[123, 196]		
*M. Soleus*					***M. Soleus***				
AT	105 ± 44	[77, 133]	0.066	0.79	AT	153 ± 51	[78, 201]	0.208	0.41
CO	144 ± 53	[110, 178			CO	130 ± 62	[90, 169]		
***M.*** ***Gastrocnemius medialis***					***M. Gastrocnemius medialis***				
AT	82 ± 28	[64, 99]	0.074	0.78	AT	143 ± 80	[92, 194]	0.434	0.34
CO	100 ± 20	[88, 113]			CO	121 ± 51	[88, 153]		
***M.*** ***Vastus medialis***					***M. Vastus medialis***				
AT	1134 ± 784	[636, 1631]	0.828	0.09	AT	250 ± 71	[205, 296]	0.933	0.04
CO	1076 ± 486	[767, 1384]			CO	254 ± 122	[176, 331]		
***M.*** ***Biceps femoris***					***M. Biceps femoris***				
AT	771 ± 303	[578, 963]	0.396	0.37	AT	169 ± 84	[116, 223]	0.464	0.31
CO	927 ± 548	[579, 1275]			CO	147 ± 55	[113, 183]		
***M.** **Gluteus maximus***					***M. Gluteus maximus***				
AT	388 ± 187	[270, 507]	0.1	0.73	AT	256 ± 149	[161, 350]	0.454	0.32
CO	576 ± 330	[366, 786]			CO	313 ± 214	[177, 449]		

### 3.7. Pain during the trials

Data for subjectively reported pain in the Achilles tendon during each trial is displayed in Table 4. Pain in the AT group ranged from 1.1 ± 1.7 to 1.9 ± 1.8, steadily rising throughout the experiment from the initial unperturbed WALK trial to the final perturbed PERTRUN task. Pain in the Achilles tendon of the CO group remained at zero throughout the experiment.

**Table 4.** Reported levels of pain during the four completed tasks.

Subjective pain rating (0–10, NRS)	AT	CO
Pain during WALK	1.1 ± 1.7	0.0 ± 0.0
Pain during PERTWALK	1.4 ± 1.7	0.0 ± 0.0
Pain during RUN	1.7 ± 1.4	0.0 ± 0.0
Pain during PERTRUN	1.9 ± 1.8	0.0 ± 0.0

## 4. Discussion

The aim of this exploratory study was to investigate neuromotor control during unperturbed and perturbed walking and running, comparing persons with AT to asymptomatic controls. The first hypothesis, that muscle activation would be early in the triceps surae and delayed in the gluteal muscles in the AT group, could only be partly accepted. The gluteus maximus musculature was significantly delayed during running, and the gastrocnemius medialis muscle showed a tendency towards earlier onset of activation with large effect sizes, although the difference between groups was not statistically significant. Hypothesis two stated that reflex latencies would be delayed in the AT group, which could only be accepted for the tibialis anterior muscle during perturbed walking and is therefore rejected for all other muscles and conditions (although many of the muscles couldn’t be analysed due to methodological issues). Finally, the third hypothesis asserted that reflex amplitudes would be reduced in the AT group, and this must be rejected as there were no statistically significant differences between the groups.

To the author’s knowledge, this is the first study to demonstrate that there is altered neuromotor control throughout the kinetic chain during both walking and running, when comparing people with AT to healthy controls. The study sheds new light on aspects of neuromuscular control in this patient group during walking, which has not been studied previously, and on neuromuscular reflex responses evoked via treadmill-induced perturbations to the lower limbs. This is a novel technique, which has not been implemented in this population in prior studies. Results from this study contribute to the body of knowledge aiming to understand AT and associated biomechanical adaptations, which may lead to improved implementation of rehabilitation and prevention strategies.

A previous study investigated the kinematics of walking in AT patients, and found that knee flexion moments at initial contact were reduced compared to a healthy control group (Kim and Yu 2015). In the current study, onset of the VM muscle prior to initial contact during walking was delayed in the AT group, indicating altered feedforward control of the knee extensors in the tendinopathic limb. The knee joint is crucial for shock absorption and stability during the initial contact of walking (Zhang et al. 2020), and the delayed onset of VM might suggest an inability of the system to control knee excursion in people with AT. Prospective research in running has shown that increased knee flexion range of motion (ROM) at both initial contact and midstance, resulted in an amplified risk of developing AT (Skypala et al. 2023). The authors suggested that increased knee flexion ROM may reduce the mechanical advantage of the gastrocnemius musculature, thus predisposing the Achilles tendon to excessive loads. However, there are two studies contradicting this theory, with results in fact reporting increased knee extension at midstance during running in people with AT (Azevedo et al. 2009; Bramah et al. 2018).

In the current study, PL muscle onset was also delayed prior to initial contact in the AT group, signalling altered feedforward control during walking. A former study found that muscle activation of the PL was lower in weight acceptance during running, in an AT population compared to healthy controls (Baur et al. 2011). The PL muscle is an ankle evertor and plays a key role in mediolateral stabilization and control of foot pronation during walking (Bavdek et al. 2018). Biomechanical research in running has suggested that overpronation might be a risk factor for developing AT (Becker et al. 2017), as excessive eversion can place repetitive high loads on the Achilles tendon (Clement et al. 1984). However, this research has yet to be reproduced in walking, and there are two studies which reported no differences in frontal and transverse plane kinematics at the ankle, in people with AT (Creaby et al. 2017; Bramah et al. 2018).

During unperturbed running, the Gmax onset was significantly delayed, and the duration of the signal shorter, in the AT group compared to CO. Additionally, the overall duration of the GM was significantly longer, mostly mediated by an earlier onset in the tendinopathic limb. These results suggest an alteration in feedforward timing of the Gmax and GM during running in people with AT. A previous study in male runners found similar outcomes in Gmax timing (Franettovich et al. 2014), and the magnitude of difference observed represented 13% of the gait cycle, whereas in the current study the differences between groups represent approximately 9% of the gait cycle. Furthermore, Chang and Kulig (2015) reported an earlier onset of GM activity in an AT group during sub-maximal hopping at a steady rate. It has been proposed that there is a direct link between hip and ankle kinetics during tasks such as walking and running, understood through the lens of kinetic chain theory (Karandikar and Vargas 2011). Altered activation in Gmax may affect the ability of the hip extensors to control movement of the femur during locomotion (Willson et al. 2011), potentially resulting in compensations distally throughout the kinetic chain, for example at the ankle (Nakashima et al. 2017). Research in walking suggests that forward propulsion is maintained by interjoint load-sharing between the hip and ankle joint, whereby an increase in the moment of one joint will result in a reduced moment at the other (Sadeghi et al. 2001; Lewis and Ferris 2008). It could also be postulated, that earlier onset in the plantar flexors produces higher moments at the ankle joint; potentially as a compensation to the impaired mechanical performance of the Achilles tendon in people with AT (Chang and Kulig 2015); thus resulting in reduced hip moments and the corresponding delayed onset signal. However, there is also evidence to suggest that the primary plantar flexors are actually inhibited in AT, showing less relative EMG activity compared to healthy controls across a range of movement tasks (Baur et al. 2011; Masood et al. 2014; Chang and Kulig 2015). It is noteworthy that the current findings only appear to manifest in the running task and are not apparent during walking. Achilles tendon forces are greater during running compared to walking (Sancho et al. 2023), and running is likely to increase demands on the calf and hip musculature (Novacheck 1998), perhaps explaining why these alterations are only then exposed. It can be surmised that the tasks of walking and running are distinct, and that the apparent neuromotor adaptations in people with AT are also distinct within each task.

The AT group exhibited significantly delayed reflex latency responses in the TA muscle during walking, indicating a potential impairment in the sensory motor feedback loop (Taube et al. 2007; Mueller et al. 2016). The TA muscle is crucial for eccentric control of the lower leg during the stance phase of walking, providing vital stability at the foot to avoid excessive dorsiflexion (Lichtwark 2014). The reactive capacity of this muscle appears impaired during walking in people with AT, perhaps as a cause or consequence of the pathological tendon. These alterations may occur due to peripheral changes in the tendon and local musculature (Arya and Kulig 2010), or perhaps due to changes in central pain processing which correspondingly impact neural reflex pathways (Hodges and Tucker 2011; Tompra et al. 2016). Previous research has shown that triceps surae reflex activity is upregulated in AT patients, though the methodologies of these studies were very different, utilising electrostimulation techniques as opposed to the more ‘organic’ methods of the current study (Wang et al. 2011; Chang and Kulig 2015). Despite the difficulties in methodological comparison, an interesting hypothesis could be formed from these findings: is the reflex excitability of a given agonist muscle, dependent upon the state of its antagonist? Perhaps in the case of AT, the upregulated triceps surae reflex inhibits antagonist reflex sensitivity in the TA. There is minimal research to support such a hypothesis, with some experimentation in the elbow joint reporting such a relationship (Villamar et al. 2023), but of course more research will be required to explore this notion.

There were no statistically significant findings for differences in reflex amplitudes, though there was a trend with medium-to-large effect sizes in the PL, Sol and GM showing reduced reflex muscle activation in response to the perturbations in the AT group during walking. These muscles are all plantar flexors, with the Sol and GM inserting directly onto the Achilles tendon. Prior studies have shown inhibited amplitudes of the GM and PL during running (Baur et al. 2011) and the Sol is also thought to be negatively impaired in AT patients (O’Neill et al. 2019). In this study, the perturbations executed during walking delivered a powerful impulse to the distal lower limb, likely resulting in large forces through the Achilles tendon. It seems plausible, that the inhibition seen in the ankle musculature, might be an attempt of the body to protect the injured/painful Achilles tendon in the AT group (Hodges and Tucker 2011). Additionally, reactive strength deficits are thought to exist in people with AT, so perhaps the reduced amplitude signal is more indicative of genuine dampening of neural drive to the corresponding musculature (McAuliffe et al. 2019). As stated, these differences were not statistically significant and should be interpreted very cautiously, but more research with larger sample sizes is warranted.

### 4.1. Limitations

Several limitations of this study should be acknowledged. First, the sample size was relatively small due to challenges in participant recruitment and strict inclusion criteria. As a result, we report effect sizes and confidence intervals to support interpretation and to inform the design of future, more robust studies. No post hoc corrections for multiple comparisons were applied due to the small sample, and thus, all findings should be interpreted as exploratory and preliminary in nature. Nevertheless, the data provide valuable insights and may serve as a foundation for further investigation. Second, the cross-sectional design prevents determination of causality; it remains unclear whether the observed neuromotor alterations are causative or a consequence of Achilles tendinopathy. Longitudinal studies with larger cohorts are recommended to address this question.

Third, walking and running speeds were standardized across participants. While this enabled consistent and repeatable perturbations at specific gait phases, it may have resulted in unnatural gait patterns for some individuals. Fourth, the walking and running tasks were not randomized, which may have introduced order effects. Furthermore, we were only able to include male runners within the current study, which limits the generalizability of the findings to female runners. Finally, the AT group was slightly older than the control group, which may have influenced group comparisons and should be considered when interpreting the findings.

## 5. Conclusions

This study reports preliminary evidence that people with AT exhibit several neuromotor alterations during the tasks of walking and running, in the lower limb musculature. These feedforward and reactive reflex alterations occur in the whole kinetic chain of the lower limb and appear to be distinct in the individual tasks of walking and running. Future research should aim to confirm or refute the findings of the current study with a sufficiently powered cohort, perhaps focusing on key muscles of interest which appear to be relevant based upon the data reported. This may assist in the development of our understanding of AT, and lead us to mechanistic models which help explain recovery during the therapeutic process. Future research should also consider investigating whether the alterations in neuromotor control in people with AT are mechanistically related to structural tendon morphology, and whether these alterations can be resolved with appropriate rehabilitation.
